# The Impact of Formal Mentorship Programs on Mentorship Experience Among Radiation Oncology Residents From the Northeast

**DOI:** 10.3389/fonc.2019.01369

**Published:** 2019-12-04

**Authors:** Mutlay Sayan, Nisha Ohri, Anna Lee, Zeinab Abou Yehia, Apar Gupta, John Byun, Salma K. Jabbour, Raquel Wagman, Bruce G. Haffty, Joseph Weiner, Sung Kim

**Affiliations:** ^1^Department of Radiation Oncology, Rutgers Cancer Institute of New Jersey, New Brunswick, NJ, United States; ^2^Department of Radiation Oncology, SUNY Downstate Medical Center, Brooklyn, NY, United States

**Keywords:** radiation oncology, education, mentorship, internship, residency

## Abstract

**Purpose:** Strong mentorship has been shown to improve mentee productivity, clinical skills, medical knowledge, and career preparation. We conducted a survey to evaluate resident satisfaction with mentorship within their radiation oncology residency programs.

**Methods and Materials:** In January 2019, 126 radiation oncology residents training at programs in the northeastern United States were asked to anonymously complete the validated Munich Evaluation of Mentoring Questionnaire (MEMeQ). Results of residents with a formal mentoring program were compared to those without a formal program.

**Results:** Overall response rate was 42%(*n* = 53). Participants were 25% post-graduate year two (PGY-2), 21% PGY-3, 26% PGY-4, and 28% PGY-5. Only 38% of residents reported participation in a formal mentoring program, while 62% had no formal program, and 13% reported having no mentor at all. Residents participating in a formal mentoring program reported strikingly higher rates of overall satisfaction with mentoring compared to those who were not (90% vs. 9%, *p* < 0.001). Overall, 38% of residents were either satisfied/very satisfied with their mentoring experience, while 49% of residents were unsatisfied/very unsatisfied.

**Conclusion:** Residents participating in a formal mentorship program are significantly more likely to be satisfied with their mentoring experience than those who are not. Our results suggest that radiation oncology residency programs should strongly consider implementing formal mentorship programs.

## Introduction

Strong mentorship has been shown to improve mentee productivity, clinical skills, medical knowledge, and career preparation (1–5). A recent survey of radiation oncology residency graduates indicated that “faculty mentorship” was the most valued factor of respondents' residency experience and that the value of mentorship extends beyond residency (6, 7). Most mentorship relationships occur via individual radiation oncology departments, though some institutions such as the American College of Radiation Oncology (ACRO) do provide some medical student/resident and resident/attending mentorship programs (8). We recently instituted a formal mentoring program at our institution based on resident feedback identifying a desire for additional mentorship.

Despite the reportedly strong correlation between mentorship and mentee success, there has been relatively little research performed regarding mentorship in radiation oncology residency. In this study, we conducted a survey to evaluate resident satisfaction with mentorship within their radiation oncology residency programs. We hypothesized that a formal mentorship program (as opposed to an informal or non-existent program) improves overall satisfaction with the mentorship experience.

## Materials and Methods

The Munich Evaluation of Mentoring Questionnaire (MEMeQ) is a validated online questionnaire of 7 items which evaluates satisfaction with mentoring relationships (9). In January 2019, 126 radiation oncology residents training at programs in the northeastern United States (Massachusetts, New Jersey, New York, and Pennsylvania) were asked to anonymously complete the MEMeQ. The survey was disseminated via an e-mail link using SurveyMonkey (Surveymonkey.com, San Mateo, CA USA) to each program's chief residents, to be forwarded to the residents presently enrolled in their training program. The survey was accessible for a 4-week period from January 1, 2019 to January 31, 2019. No personal information was collected. This study was conducted according to the Rutgers Cancer Institute of New Jersey institutional review board guidelines. Responses were compared based on reported participation in a formal vs. less than formal mentoring program. Univariate comparisons were performed using Chi-squared tests.

## Results

Overall response rate was 42% (*n* = 53). Participants were 25% post graduate year two (PGY-2), 21% PGY-3, 26% PGY-4, and 28% PGY-5. Thirty-one residents (59%) reported having one or two mentors, and seven residents (13%) reported having no mentor. The top three areas where mentoring was found to be helpful included research (94%), job opportunities (92%), and networking (84%). Other areas mentioned included guidance on public speaking/presentation (35%) and work-life balance (13%).

Mentors were described as approachable (personality, manner) by 68% of residents, supportive/encouraging by 65%, providing guidance on course of study or career management by 60%, answering questions satisfactorily (e.g., timely, clear, comprehensive) by 60%, motivating by 53%, and accessible by 49%. Overall, 38% of the residents were satisfied with their mentoring experience while 49% were dissatisfied and the remainder were ambivalent (Table 1). On further analysis, we found that 38% of PGY-2s, 45% of PGY-3s, 35% of PGY-4s, and 33% of PGY-5s are satisfied with the mentorship.

**Table 1:** Descriptive responses from the adapted MEMeQ of residency mentorship experience in radiation oncology.

**Feature**	**Number (%)**
Year in training	
PGY-2	13 (25%)
PGY-3	11 (21%)
PGY-4	14 (26%)
PGY-5	15 (28%)
Formal mentorship program	
Yes	20 (38%)
No	33 (62%)
Number of mentors	
0	7 (13%)
1	20 (38%)
2	11 (21%)
3	11 (21%)
≥4	4 (8%)
Mentorship characteristics	
Is approachable	32 (68%)
Is supportive and encouraging	30 (65%)
Answers my questions satisfactorily	28 (60%)
Provides direction and guidance regarding my course of study or career management	28 (60%)
Motivates me to reach my objectives	25 (53%)
Is accessible	23 (49%)
Satisfaction with areas of interest	
Site-specific expertise	26 (62%)
Research	23 (49%)
Networking	15 (38%)
Job opportunities	16 (36%)
Work-life balance	12 (32%)
Global health	4 (11%)
Overall satisfaction	
Very unsatisfied	10 (19%)
Unsatisfied	16 (30%)
Neither	7 (13%)
Satisfied	11 (21%)
Very satisfied	9 (17%)

*MEMeQ, Munich-Evaluation-of-Mentoring-Questionnaire; PGY, post-graduate year*.

Twenty residents (38%) reported participation in a formal mentoring program with regular interval meetings. Residents participating in a formal mentoring program reported significantly higher rates of overall satisfaction with mentoring compared to those who were not (90% vs. 9%, *p* < 0.001). Mentors' accessibility (94% vs. 27%, *p* < 0.001) and ability to answer questions satisfactorily (100% vs. 69%, *p* = 0.012) also improved with formal mentoring programs (Table 2).

**Table 2:** Resident reported satisfaction in programs with and without formal mentorship curriculums.

	**Formal mentorship**		***p***
	**Yes (%)**	**No (%)**	
Satisfied with mentoring	90	9	<0.001
Mentorship characteristics:			
Is accessible	94	27	<0.001
Is supportive and encouraging	100	92	0.263
Answers my questions satisfactorily	100	87	0.157
Provides direction and guidance	100	92	0.259
Is approachable	100	83	0.070
Motivates me to reach my objectives	100	69	0.012

## Discussion

Our study addresses the impact of structured mentorship curriculums in radiation oncology residency programs. To our knowledge, this is the first study assessing radiation oncology residents' satisfaction with mentorship using a validated questionnaire. In a heterogeneous cohort of radiation oncology residents, we noted two main findings: nearly 50% of participating residents reported overall dissatisfaction with mentorship during residency; and residents participating in a formal mentorship program were significantly more likely to be satisfied with their experience.

Multiple other studies have validated the need for and effectiveness of faculty mentoring. In 2014, a nationwide survey was disseminated to evaluate factors predictive of having a mentor and satisfaction with the mentorship experience in radiation oncology residency (10). In this study with 150 responses (25% response rate), a majority of residents (85%) reported that mentorship plays a critical role in residency training and career development, and most of the residents (74%) reported a desire to participate in a formal mentorship program. Furthermore, a formal mentorship program was associated with increased satisfaction with the mentorship experience. Ko and Kimple recently reported their experience instituting a formal program for trainees to regularly assess career goals with their mentors (7). In this study, a formal resident individual development plan significantly increased residents' confidence in achieving career goals, having a plan to develop strengths, and bolstered the mentor-mentee relationship. Furthermore, a nationwide survey reported that approximately one-third of radiation oncology residents have high levels of burnout symptoms (11). Potentially, regular meetings in a formal mentoring curriculum could help identify residents at high risk of burnout and lead to early intervention.

Our study's high response rate of 42% indicates residents' high interest in faculty mentorship. The limitations of our study include its small sample size, absence of demographic information such as age, marital or parental status, and that the survey was conducted exclusively in the Northeast, as opposed to the entire US. Compared to previous work, the strength of our study was the utilization of a validated questionnaire to determine residents' satisfaction with mentoring relationships. In conclusion, we found that residents within a formal mentorship program were much more satisfied (90% vs. 9%) with their mentorship experience. Given the proven importance of mentoring in terms of resident satisfaction and future success, our results suggest that radiation oncology residency programs should strongly consider implementing formal mentorship programs if they have not already.

## Data Availability Statement

All datasets generated for this study are included in the article/supplementary material.

## Ethics Statement

The studies involving human participants were reviewed and approved by Rutgers Cancer Institute of New Jersey. Written informed consent for participation was not required for this study in accordance with the national legislation and the institutional requirements.

## Author Contributions

All authors listed have made a substantial, direct and intellectual contribution to the work, and approved it for publication.

### Conflict of Interest

The authors declare that the research was conducted in the absence of any commercial or financial relationships that could be construed as a potential conflict of interest. The handling Editor declared a past co-authorship with several of the authors SJ and SK.
